# Routine panendoscopy in oral squamous cell cancer patients: mandatory or facultative?

**DOI:** 10.1007/s00784-020-03429-8

**Published:** 2020-07-01

**Authors:** Anthony Valentin, Martin Goetz, Juergen Hetzel, Siegmar Reinert, Sebastian Hoefert

**Affiliations:** 1 Department of Oral and Maxillofacial Surgery, University Hospital of Tuebingen, Osianderstr. 2-8, 72076 Tuebingen, Germany; 2 Department of Internal Medicine, Hospital of Sindelfingen-Boeblingen, Bunsenstr. 120, 71032 Boeblingen, Germany; 3 Department of Molecular Medicine and Pneumology, University Hospital of Tuebingen, Otfried-Mueller-Str. 10, 72076 Tuebingen, Germany

**Keywords:** Oral cancer, Routine panendoscopy, Synchronous second primary cancer, Gastrointestinal inflammation, National guidelines

## Abstract

**Objectives:**

This study investigated benefits of routine panendoscopy in staging of oral squamous cell cancer patients.

**Materials and methods:**

From 2013 to 2017, 194 oral squamous cell cancer patients were staged. Reports of routine flexible panendoscopy including oropharyngolaryngoscopy, bronchoscopy, and esophagogastroduodenoscopy were retrospectively analyzed for diagnoses of inflammation and second primary malignancies (carcinoma in situ or cancer) and compared to results of computed tomography. The effects of alcohol and tobacco history of 142 patients were assessed.

**Results:**

Overall, a second primary malignancy was detected in seven patients. In four patients this discovery was only found by panendoscopy. One invasive carcinoma (esophagus) was detected as well as three carcinoma in situ. The second primary malignancies were located in the lung (3), esophagus (3), and stomach (1). In one patient index tumor therapy was modified after panendoscopy. Upper gastrointestinal inflammation was present in 73.2% of patients and 61.9% required treatment. About 91.8% of bronchoscopies and 34.5% of panendoscopies were without therapeutic consequences. Patients with higher risk from smoking were more likely to benefit from panendoscopy and to have a Helicobacter pylori infection.

**Conclusion:**

We do not recommend routine panendoscopy for all oral squamous cell cancer patients. Esophagogastroduodenoscopy benefitted smoking patients primarily concerning the secondary diagnosis of inflammation of the upper digestive tract. Selective bronchoscopy, esophagogastroduodenoscopy, and oropharyngolaryngoscopy should be performed if clinical examination or medical history indicates risks for additional malignancies of the upper aerodigestive tract.

**Clinical relevance:**

Routine panendoscopy is not recommended in all, especially not in low-risk oral cancer patients like non-smokers and non-drinkers.

## Introduction

In Europe, 5–10% of new cancer cases are head and neck cancers [1]. Over 90% of head and neck cancers are squamous cell carcinoma [2, 3]. They mostly are located in the upper aerodigestive tract, with oral cavity making up 44%, larynx 31%, and pharynx 25% of squamous cell carcinoma [3].

Billroth was the first to describe multiple distinct simultaneous cancers [4]. A definition of multiple primary cancer was presented by Warren and Gates and states that the tumors must be malignant, distinct, and without the possibility of them being metastases [5]. Slaughter proposed the concept of “field cancerization” [6]. It supposed that a noxa exerts a cancerogenic effect on the entire contacted susceptible tissue. Thus, multiple distinct malignant lesions may develop at the same time [6]. Two noxae were identified as alcohol and tobacco, which temporarily accounted for 75% of oral cancers in the USA [7]. While some studies found more second primary cancers in patients with floor of mouth as index tumors [8–11], others were unable to confirm their results [12].

Following research on multiple primary cancers in head and neck cancer patients, routine panendoscopy including oropharyngolaryngoscopy, bronchoscopy, and esophagoscopy was recommended as standard staging protocol for head and neck cancer patients [13, 14]. While routine panendoscopy still is standard procedure in Germany [15, 16], its indication has been put up for debate.

Proponents of standard panendoscopy have referred to its benefit towards identifying second primary malignancies, thus changing clinical strategy and potentially preventing the progression of early stage second primary cancer [17–20]. Opponents have emphasized the cost, time delay of definite therapy, and low yield of second primary cancer of bronchoscopy or esophagoscopy over other staging procedures, like clinical examination and radiologic imaging technologies [9, 10, 13, 21–24]. The widespread availability and qualitative improvements of computed tomography (CT), magnetic-resonance imaging (MRI), and positron-emitting tomography (PET) have increased the diagnostic sensitivity of imaging. Oropharyngolaryngoscopy may suffice for planning of therapy and tissue sampling, and routine panendoscopy may not be required for adequate staging in every head and neck cancer patient [23, 25].

The incidence of synchronous second primary cancers in head and neck cancer patients has been reported with a range of 1–30% [13, 26, 27]. The German guidelines for diagnosis and treatment of oral cavity carcinoma state synchronous second primary cancer and distant metastases were prevalent in 4–30% of patients with oral cavity squamous cell carcinoma [15]. Furthermore, routine staging panendoscopy should be performed in all patients [15]. The requirements for German clinics to be certified as head and neck cancer center demand routine panendoscopy of every patient with squamous cell head and neck cancer [16].

In a single head and neck cancer center, we wanted to assess the diagnostic relevance of routine panendoscopy in staging of head and neck cancer patients treated between 2013 and 2017. Approval was granted by the ethics committee of the University of Tuebingen (IRB: 707/2017B02).

## Subjects and methods

**Fig. 1:**
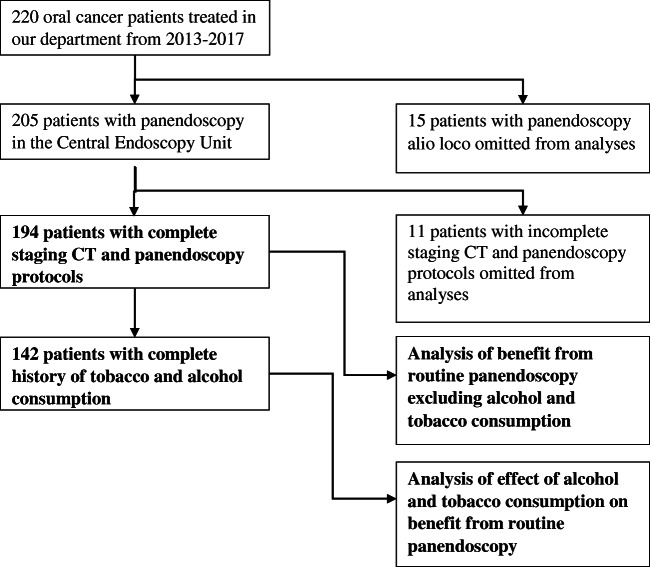
Inclusion criteria for this study and for inclusion in analysis of benefit from panendoscopy and analysis of effect of alcohol and tobacco consumption

From 2013 to 2017, 220 head and neck squamous cell cancer patients were treated in our clinic, of which 194 completed all staging procedures in our center and included in our study (Fig. 1). Staging included CT of head, neck, thorax, and abdomen prior to panendoscopy. Panendoscopy was performed by flexible endoscopes during sedation and included oropharyngolaryngoscopy, bronchoscopy, and esophagogastroduodenoscopy (EGD). The panendoscopies were performed within 14 days of index tumor diagnosis by the Department of Interdisciplinary Endoscopy of the University Hospital of Tuebingen. Evaluation of oropharyngolaryngoscopy was included in bronchoscopy reports, if applicable. A total of 194 patients received a complete panendoscopy and entered analysis of diagnostic yield of routine panendoscopy.

Staging of disease and index tumor location were defined according to the 7th Edition of the UICC [28]. As per the TNM classification, index tumor location was divided into oropharyngeal cancers, oral cavity and lip cancers, and extraoral head and neck squamous cell cancers of unknown primary (CUP). Tumors of the hypopharynx and larynx were not encountered in our study sample. We further distinguished floor of mouth cancers from cancers of the remaining oral cavity.

Carcinoma in situ and invasive cancers identified through panendoscopy were summed up as synchronous second primary malignancies (SPM). Gastrointestinal and bronchial inflammation were evaluated from panendoscopy reports.

The degree of gastrointestinal inflammation was evaluated: none being lowest, followed by discrete, moderate, atrophic, erosive, and ulcerative being highest. Helicobacter pylori (HP) status and reports of suspicious lesions were analyzed. Therapeutic consequences were categorized by no consequences, control EGD, medication (proton pump inhibitors (PPI), antimycotics, HP eradication), excision, and change of index tumor therapy by the tumor board.

The degree of bronchial inflammation was evaluated (normal, atrophic, vulnerable, bronchitic, COPD-like, anthracosis), as were reports of suspicious lesions. Therapeutic consequences were categorized by sectional imaging, ENT-assessment, antibiotic treatment, tuberculosis diagnostics, biopsy, excision/resection, and change of index tumor therapy by the tumor board.Histopathological results were checked for degree of malignancy (none, carcinoma in situ (CIS), cancer), and the positive predictive value (PPV) for malignancy of suspicious lesions was calculated. We compared the diagnostic yield of routine panendoscopy over staging CT of head, neck, thorax, and abdomen for the detection of synchronous primary malignancies.

Per the German guidelines, low-risk alcohol consumption was defined as alcohol consumption of equal or less than 120 g/week for men and 60 g/week for women respectively, and more as high-risk alcohol consumption [29]. Previous high-risk alcohol consumption was categorized as ex-high risk. Risk from alcohol consumption was ranked from none (0), low risk (1), ex-high risk (2) to high risk (3).

Similar to Leoncini et al. [30], we defined heavy smokers as those with more than 20 pack years or more than 20 cigarettes per day and light smokers as smokers consuming less. Risk from tobacco consumption was graded from none (0), ex-smoker (1), light smoker (2) to heavy smoker (3).

We correlated alcohol and tobacco consumption with age and level of inflammation in the upper digestive tract using Spearman’s rho. We compared men and women regarding their risk from alcohol and tobacco consumption using a Mann–Whitney *U*- test (MWU). HP-positive and HP-negative patients were compared regarding their risk from alcohol and tobacco consumption using an MWU.

Two separate analyses were conducted. In Analysis 1 (A1) patients with SPM and patients without SPM were compared. In Analysis 2 (A2) patients with and patients without therapeutic consequences from panendoscopy were compared. In both analyses, age was compared using an independent *t* test. Sex was compared using Fisher’s exact test in A1 and Pearson’s chi-square test in A2. Index tumor location was compared using likelihood-ratio chi square test. Tumor stage was analyzed using an MWU.

For cases with complete alcohol and tobacco history, alcohol consumption and intensity of smoking were analyzed using an MWU in both analyses.

Statistic results were rated significant at the 0.05 level.

## Results

A total of 194 patients with oral squamous cell carcinoma treated in our clinic from 2013 to 2017 were included in our study. Of these 66.5% were male and 33.5% female. Age ranged from 32 to 94 years with a mean of 63.3 ± 12.4 years. Index tumor location varied between 34% floor of mouth, 60.3% for lips and remaining oral cavity, 5.2% for oropharynx, and 0.5% (1 case) for CUP (Table 1). About 42% of patients presented with early stages of disease I–II and 58% with advanced stages III–IVa/b/c. In 0.5% (1 case) distant metastasis was diagnosed.

**Table 1:** Distribution of index tumor locations and synchronous second primary malignancies (SPM) in this study

Tumor location	Patients	Patients with SPM	Quota^a^	Lung SPM	Digestive tract SPM
Floor of mouth	66 (34%)	5 (71.4%)	7.6%	2 cancers, 1 CIS	1 cancer esophagus, 1 CIS stomach
Lips and remaining oral cavity	117 (60.3%)	1 (14.3%)	1.7%	0	1 Cancer esophagus
Oropharynx	10 (5.2%)	1 (14.3%)	10%	0	1 CIS esophagus
CUP	1 (0.5%)	0	0%	0	0
	Total: 194 (100%)	Total: 7 (100%)			

^a^Percentage of patients with common index tumor location that had a synchronous second primary malignancy (SPM)

### Results of panendoscopy

Four out of 7 SPM including all 3 CIS were only detected via panendoscopy. Three out of 4 synchronous invasive cancers were also detected by prior radiologic imaging. The yield of panendoscopy over imaging was 2.1% (4 cases) for SPM and 0.5% (1 case) for synchronous second primary invasive cancers (Table 2). Therapy of the index tumor was modified in only 0.5% (1 case), where the adjuvant radiotherapy was extended to include the SPM site in addition to the index tumor site. Panendoscopy did not have any therapeutic consequences in 34.5% of all patients, and in 25.4% of them, biopsies were performed with benign results.

**Table 2:** Patients with synchronous second primary malignancies (SPM)

Patient	P1	P2	P3	P4	P5	P6	P7
Sex	Male	Male	Male	Male	Male	Male	Female
Age	73	66	80	71	52	57	48
Index tumor location	Floor of mouth	Lips and remaining oral cavity	Floor of mouth	Floor of mouth	Floor of mouth	Oropharynx	Floor of mouth
Stage^a^	IVa	II	IVa	I	II	IVa	IVa
Alcohol^b^	High risk	No	Low risk	No	Low risk	Ex-high risk	Ex-high risk
Tobacco^b^	Ex-smoker (45 PY)	Heavy smoker	Ex-smoker	Light smoker	Light smoker	Ex-smoker	Heavy smoker
Inflammation in upper digestive tract^c^	Ulcerative	Atrophic	Moderate	None	Ulcerative	Discrete	Discrete
HP^d^	Negative	Negative	Negative	Negative	Positive	Negative	Negative
Bronchial aspect^e^	Chronic bronchitis	Chronic bronchitis	COPD-like	Bronchitis	COPD-like	Normal	COPD-like
Synchronous second primary malignancy	CIS distal corpus of stomach	Squamous cell cancer of esophagus	Non-small cell cancer of lung T1b N2-3 M0	Bronchial cancer pT1a	Squamous cell CIS of lung	CIS of esophagus	Squamous cell cancer of esophagus
Diagnosed with staging CT prior to panendoscopy	No	No	Yes	Yes	No	No	Yes
Second primary malignancy therapy	Excision	Radiotherapy	Supportive care	Surgery	Excision	Excision	Surgery
Change of index tumor therapy^f^	No (surgery and RT^g^)	Extension of adjuvant RT of index to SPM sites	No (RT)	No (surgery)	No (surgery)	No (surgery and RT)	No (adjuvant RT of index site)

^a^Stage of disease (“Subjects and methods”)

^b^Alcohol and tobacco risk levels (“Subjects and methods”)

^c^As present at panendoscopy, ranked from none to ulcerative (“Subjects and methods”)

^d^Helicobacter pylori status

^e^Aspect of bronchial mucosa and bronchi at panendoscopy

^f^Index tumor therapy given in brackets, if no change occurred

^g^Radiotherapy

EGD reports identified suspicious lesions in 11.9% of 194 patients. Histopathologic analysis revealed 2 CIS, 2 cancers, and 19 benign results (PPV 17.4%). In 2.1% of patients (4 cases) a second primary malignancy could be detected via EGD. Two of them were CIS; the other two were invasive cancer. The prevalence of inflammation in esophagus, stomach, or duodenal bulb was 73.2%. About 32% of patients had ulcerative inflammation, 10.3% erosive lesions, 3.1% (6 cases) atrophic gastritis, 17% moderate signs of inflammation, and 10.8% discrete inflammation within the upper gastrointestinal tract. In 58.2% samples were taken. HP test was positive in 11.3%. In 34.5% control EGD was recommended. In 61.9% medication (PPI, antimycotics, or HP eradication therapy) was recommended. In 25.8% of all cases the only consequence of EGD was prescription of PPIs. In 1% (2 cases) of all cases antimycotics were prescribed. In 0.5% (1 case) an excision was planned, and in 1% (2 cases) the case should be presented to a gastrointestinal tumor board. There was no therapeutic consequence of EGD in 37.1% of patients.

Bronchoscopy reports described suspicious lesions in 8.8% of 194 patients. Samples were taken in 8.2%. In 1.5% (3 cases) a second primary malignancy was found: one of them CIS and the other two cancers. The remaining 14 were benign (PPV 17.6%). Results of bronchoscopy showed an abnormal bronchial aspect in 36.6%. Bronchial aspect was described as atrophic in 4.1% (8 cases) of patients, as vulnerable in 1% (2 cases), as bronchitic in 8.2%, as COPD-like in 21.6%, and as including anthracosis in 1.5% (3 cases) of patients. In 0.5% (1 case) further sectional imaging was recommended. In 1.5% (3 cases) the patient should be assessed by an ENT-specialist. In 2.1% (4 cases) antibiotic treatment of an infection was prescribed. In 0.5% 1 (1 case) tuberculosis diagnostics were recommended. In 0.5% (1 case) an excision or resection of a lesion was planned, and in 1.0% (2 cases), the cases were presented to a lung tumor board afterwards. In 91.8% there was no therapeutic consequence of bronchoscopy.

### Alcohol and tobacco history

Alcohol and tobacco consumption information was complete for 142 patients (Fig. 1). About 56.4% were non-drinkers, 9.3% low risk drinkers, 12.1% ex-high risk drinkers, and 22.1% high risk drinkers. About 40.7% were non-smokers, 9.3% ex-smokers, 27.1% light smokers, and 22.9% heavy smokers. About 33.8% of patients were non-drinkers and non-smokers, and 13.4% of patients were high-risk drinkers and heavy smokers.

Alcohol and tobacco consumption were correlated (Spearman’s rho = 0.57, *p* < 0.01). With increasing age, patients engaged in less risk from alcohol or tobacco consumption (alcohol: *p* < 0.01; tobacco: *p* < 0.01). Men engaged in riskier alcohol and tobacco consumption than women (alcohol: *p* = 0.01; tobacco: *p* < 0.01). Level of inflammation in the upper digestive tract correlated positively with risk from alcohol (*p* = 0.02) and tobacco consumption (*p* < 0.01) (Table 3).

**Table 3:** Distribution of level of inflammation in the upper digestive tract by risk from tobacco consumption and Helicobacter pylori (HP) status

	Inflammation of the upper digestive tract^a^						
Risk from tobacco consumption	None	Discrete	Moderate	Atrophic	Erosive	Ulcerative	Total (%)
HP positive							
None	0	1	0	0	0	2	3 (18.8)
Ex-smoker	0	0	0	0	0	0	0 (0)
Light smoker	0	0	1	0	1	4	6 (37.5)
Heavy smoker	0	0	0	0	0	7	7 (43.8)
Total (%)	0 (0)	1 (6.3)	1 (6.3)	0 (0)	1 (6.3)	13 (81.3)	16 (100)
HP negative							
None	21	6	11	1	2	13	54 (42.9)
Ex-smoker	3	1	4	1	2	3	14 (11.1)
Light smoker	9	3	9	2	3	7	33 (26.2)
Heavy smoker	4	4	1	1	7	8	25 (19.8)
Total (%)	37 (29.4)	14 (11.1)	25 (19.8)	5 (4.0)	11 (8.7)	28 (22.2)	126 (100)

^a^Values represent cases. Only patients with complete tobacco and alcohol history were included (142 from 194 total)

Alcohol and tobacco consumption history was complete for 73.3% of HP-negative and for 72.3% of HP-positive patients. Patients testing positive for HP had higher associated risk of gastrointestinal inflammation from tobacco consumption than patients testing negative for HP (*p* = 0.01). They did not differ in risk from alcohol consumption (*p* = 0.64) (Table 3).

### A1: Analysis of patients with versus patients without synchronous primary malignancy

About 3.6% (7 cases) had SPM identified by panendoscopy (Table 2), including 3 CIS (1.5%; lung 1, esophagus 1, stomach 1) as well as 4 invasive cancers (2.1%; lung 2, esophagus 2). Six patients were men with a mean age of 66.5 (10.4), and one was a woman of 48 years. About 96.4% of patients were without SPM. Of these, 65.8% were male with a mean age of 62.3 (12.1), and 34.2% were female with a mean age of 65.1 (13.2). No SPM of the head and neck was found. About 71.4% of patients with SPM had a floor of mouth index tumor.

Groups did not differ in age, sex, index tumor location, stage of disease, and alcohol or tobacco consumption (all *p* ≥ 0.20).

### A2: Analysis of patients with versus patients without therapeutic consequences from panendoscopy

Of the patients without therapeutic consequences from panendoscopy, 52% were male and 48% female. Age ranged from 35 to 94 years with a mean age of 66.1 (13.9). About 64% were non-drinkers, 10% low risk, 14% ex-high risk, and 12% high risk alcohol consumers. About 54% were non-smokers, 10% ex-smokers, 26% light smokers, and 10% heavy smokers. About 46% were non-drinkers and non-smokers, and of them 73.9% were female.

Groups did not differ in age, index tumor location, stage of disease, or alcohol consumption (all *p* ≥ 0.07). Women were less likely to have therapeutic consequences from panendoscopy than men (*p* < 0.01), with an effect size of Cramer’s V = 0.22. Risk from tobacco consumption was significantly higher in patients with therapeutic consequences from panendoscopy than in patients without (*p* < 0.01).

## Discussion

In this study of patients with oral squamous cell carcinoma, the rate was 2.1% (4 cases) for synchronous primary cancer and 0.5% (1 case) for distant metastasis of the index tumor. All SPM were found in the lung or upper gastrointestinal tract. This is lower than the 4–33% for second primary cancer and distant metastasis suggested by the German guidelines for diagnosis and treatment of oral cavity carcinoma [15]. If second primary carcinoma in situ lesions found in 1.5% (3 cases) in this study are added to synchronous primary cancer and distant metastasis, we reach a total rate of 4.1% (8 cases) of patients with synchronous second primary malignancies or distant metastasis. The studies referenced in the guideline report frequencies of synchronous second primary cancer and distant metastasis ranging 0–15.9%, with a mean of 4.6% (5.5%) [15, 31, 32]. The study with the highest rate of 15.9% second primary cancer was published by Lee et al. in 2010 [31]. They found esophageal second primary neoplasia in 30.4% of patients with head and neck index tumors, of which 76.2% were synchronous. No bronchoscopy results were reported. It is noteworthy that almost half of the patients who were included in the second primary neoplasia rate had early stage noninvasive neoplasia [31]. Furthermore, regional factors of Taiwan not found in Western Europe are linked to an elevated risk of esophageal and gastric cancers [33, 34].

It has been debated whether patients with SPM have any benefit in quality of life or survival from the detection of their SPM at time of staging of the index tumor [25, 35–37]. Index tumor therapy was only modified in 0.5% (1 case), being additional adjuvant radiotherapy of the SPM site. In one case, synchronous second primary cancer was treated only with supportive care as the patient did not wish to undergo further therapeutic load and radiotherapy of the index tumor was not successful. While long-term survival of patients with SPM was not part of this study, the SPM identified were treated curatively in all but one case.

We encountered a high rate of 73.2% patients with inflammation of the upper digestive tract. Compared with the results from Kesting et al., patients in our sample were more than twice as likely to have inflammation of the esophagus, stomach, or duodenal bulb (73.2% to 31%) [38]. Asymptomatic gastro-esophageal reflux disease is common within head and neck cancer patients [3]. Estimation state half of the global population has chronic gastritis. Main contributor is HP infection (90%). Severe atrophic gastritis and acid-free stomach has a high risk of stomach cancer. Eradication of HP decreases the risk of ulcers and may prevent stomach cancer [39]. Between 2009 and 2016, estimated prevalence of HP infections in Germany has increased to 35%, with the mean being at approximately 18%. Estimated European infection rate is 35% and ranges from 15 to 50% [40].

From 1999 to 2014 overall incidence of oral squamous cell carcinoma in Germany decreased, especially for men and people younger than 60 [41]. Under the presumption that our patient sample was representative for Western Europe, we would not recommend routine panendoscopy in staging of all patients with oral squamous cell carcinoma. The rate of synchronous second primary cancers was a lot lower than in older studies referenced in the German guidelines [15]. The yield of panendoscopy was 0.5% for invasive cancers over radiologic imaging. This corroborates findings of similar low yield in earlier studies [23, 42–44]. Non-smokers and non-drinkers are significantly less likely to have therapeutic consequences from routine panendoscopy. We suggest that this group should be exempted from routine panendoscopy. Since women in this study exerted less riskier consumption of alcohol and tobacco than men, this may explain why they were less likely to have therapeutic consequences from panendoscopy. Rate of HP infections and ulcerative inflammation of the upper gastrointestinal tract resulting at least in new medication increased with risk from smoking. Therefore, we recommend that patients with higher risk from smoking and squamous cell oral cancer should undergo an EGD. In over 90% of patients, bronchoscopy did not have any therapeutic consequence. It is to be noted that the staging CT images were available to the staff conducting the panendoscopy prior to the procedure. This may have inflated the detection rate of SPM via bronchoscopy, as CT has proven to be more reliable in detecting secondary lesions. We would not recommend bronchoscopies as part of routine staging procedures. Bronchoscopy should be reserved to gather tissue samples from suspicious lesions detected via imaging or when clinical examination and medical history warrant it [24, 43, 45, 46].

We support the approach by other European countries, which have already left routine panendoscopy in favor of “symptomatic endoscopy” of suspicious lesions suspected from patient history or detected by prior radiologic imaging [43, 44, 46, 47]. Among them are the Netherlands, the UK, and France. Of these, only in France is routine esophagoscopy still recommended in staging of all patients with cancers in the aerodigestive tract. All four do not recommend routine bronchoscopies [43, 44, 46, 47]. Except for the French guideline, they resemble the guidelines published by the National Comprehensive Cancer Network for the USA [45], which recommends only oropharyngolaryngoscopy as routine procedure for head and neck cancer patients.

No risk factors for the development of synchronous second primary cancers in oral cancer patients were identified. This may be attributed to the small sample size. Advances in technology allow for a better understanding of the genetic and pathophysiology of field cancerization and may incite risk adapted procedures for high risk patients with pre-malignant lesions in the future [48]. Further research using larger sample sizes or meta-analysis is needed to reveal traits or risk factors that increase the risk of SPM. Staging procedures may be individualized once these are discovered.

An update to the German guidelines for oral cavity cancer is in progress assessing, e.g., the importance of HPV-16 status [49]. An HPV-16 status is required for oropharyngeal cancer staging according to the 8th edition of the TNM Classification of Malignant Tumors by the UICC. As of 1990, human papilloma virus (HPV) infections have contributed increasingly to the development of oral cancers in the USA, while the role of alcohol and tobacco has declined [3, 50]. With the increase of HPV-caused oral cancers, incidence of second primary cancers in head and neck cancer patients in the USA has declined [50, 51]. It is estimated that currently 25% of oropharyngeal cancers worldwide are caused by HPV-infections, 90% of them by the subtype HPV-16 [52]. In our patient population HPV-16 diagnosis was not routinely performed, so we cannot corroborate whether the low incidence of SPM in our study may be related to a high contribution of HPV to index tumor development. Multicontinental studies from 2004 to 2009 found that alcohol and tobacco caused 72% of head and neck cancers worldwide, but only 51% in North America [53]. Although the traditional factors tobacco and alcohol for head and neck cancer development appear to be less dominant than 30 years ago, they are still a contemporary issue [3, 53]. According to the Global Information System on Alcohol and Health by the WHO, German average daily alcohol intake in 2016 excluding the abstinent was estimated to be 51.9 g/day for men and 18 g/day for women, far more than what German low-risk alcohol consumption guidelines recommend [29]. A recent survey of over 70,000 Germans showed that 14.9% were regular smokers and 1.5% heavy smokers (more than 20 cigarettes per day) [54]. Even though we could not find a correlation between synchronous SPM and alcohol consumption like Leoncini et al. [30], the effects of high alcohol and tobacco consumption on other organ systems and the documented contribution to the development of primary head and neck cancers will continue to play a major role for doctors in the decades to come [53, 55–57].

## Conclusion

The rate of second primary malignancy was lower than previously reported in the literature cited by the German guidelines for diagnosis and treatment of oral cavity carcinoma [15]. We do not recommend routine panendoscopy in staging for all primary oral squamous cell cancer patients. Staging panendoscopy is not recommended for low-risk patients like non-smokers and non-drinkers. Smoking patients profited from an EGD, especially concerning the secondary diagnosis of inflammation of the upper digestive tract. Selective bronchoscopy, esophagogastroduodenoscopy, and oropharyngolaryngoscopy should be performed if clinical examination or medical history indicates risks for additional malignancies of the upper aerodigestive tract [43, 44, 46, 47]. This includes high-risk smokers and drinkers. Additionally, they can be used for histological sampling of suspicious lesions detected via imaging.
